# Adverse event reporting and regulatory analysis of pediatric cardiac assist devices

**DOI:** 10.1371/journal.pone.0355401

**Published:** 2026-08-12

**Authors:** Songyining Ding, Susmitha Wunnava, Gaite Liu, Hongyi Yu, Sheng Hu, Yaohua Li, Xinyan Zhang, Peiming Zhang, Yunzhang Cheng, Florence T. Bourgeois, Xun Liu

**Affiliations:** 1 School of Health Science and Engineering, University of Shanghai for Science and Technology, Shanghai, China; 2 Harvard-MIT Center for Regulatory Science, Harvard Medical School, Boston, Massachusetts, United States of America; 3 Shanghai Institute of Medical Device Testing, Shanghai, China; 4 Yangtze River Delta Center for Medical Device Evaluation and Inspection of NMPA, Shanghai, China; 5 Computational Health Informatics Program, Boston Children’s Hospital, Boston, Massachusetts, United States of America; 6 NMPA Center for Innovation and Research in Regulatory Science, Shanghai, China; CUNY School of Medicine: The City College of New York CUNY School of Medicine, UNITED STATES OF AMERICA

## Abstract

**Background:**

Due to differences in anatomy, coagulation and neurodevelopment, the complication rate of pediatric cardiac assist devices during use is much higher than that of adults, which cannot be directly extrapolated from adult data. Moreover, there are deficiencies in the device supervision process.

**Method:**

In this study, 280 adverse events of patients under 18 years old and 1,231 adverse events of adult patients were extracted from the FDA MAUDE database from 2015 to 2025. The pediatric complication spectrum was systematically analyzed, and adult data was compared with pediatric data. Combined with literature and typical case analysis, the limitations of traditional databases were jointly revealed. Finally, in accordance with the existing testing standards, the risks exposed after the product is launched on the market are summarized and the regulatory loopholes are revealed.

**Result:**

MAUDE pediatric reports are scarce and variables are missing, making it impossible to directly calculate the incidence rate. The research calls for the establishment of a multi-source real-time monitoring and pediatric exclusive registration system to preliminarily assess and generate hypotheses regarding the risk of mechanical circulatory support in children.

**Conclusion:**

This study provides a descriptive evaluation of reporting trend disparities between pediatric and adult cohorts. Given the inherent constraints of passive surveillance data, these exploratory findings should be interpreted as a reference to guide future prospective evaluations and standard optimizations, rather than immediate, broad regulatory mandates.

## Introduction

Facing life-threatening end-stage heart failure and cardiogenic shock, the treatment of pediatric patients heavily relies on cardiac assist devices, such as Intra-aortic Balloon Pump(IABP), Extracorporeal Membrane Oxygenation (ECMO), and Ventricular Assist Device (VAD). These devices can partially or completely replace the heart’s pumping function, maintain systemic circulation, reduce cardiac load, and improve myocardial oxygen supply, providing life support for children [1]. However, the rapidly changing anatomical structures, developing nervous system, and coagulation function in children make device adaptation complex. Direct application of adult devices and techniques presents significant adaptation challenges and directly leads to unique and higher risks of complications.

The core regulatory conflict in pediatric cardiology stems from the disparity between the clinical need for cardiovascular devices in children and the high risk of complications: the gap between children’s urgent need for safe and effective treatment devices and the insufficient support from the existing regulatory evidence system. This conflict obscures the true risk profile of the pediatric population. This has led to a long-standing, vague, and potentially underestimated understanding of the safety of pediatric cardiac assist devices [2,3]. Therefore, systematically revealing the true differences in adverse events between pediatric and adult patients, scientifically evaluating the limitations of existing regulatory databases, and revealing the risk lag of the existing detection standards by combining real-world data become the empirical basis for promoting precise regulation of pediatric medical devices.

This study, based on the FDA MAUDE database, focuses on three types of devices: IABP, VAD, and ECMO. By systematically analyzing pediatric adverse event data and comparing their main complications with adult data, this research aims to quantify the risk differences and empirically assess the effectiveness and shortcomings of traditional regulatory data in pediatric applications. This will provide crucial evidence for building a life-cycle regulatory framework for pediatric medical devices based on real-world evidence.

## Research methods

This study employed a comprehensive data collection and analysis approach. It collected data on adverse events related to the use of cardiac assist devices in juvenile patients from regulatory databases, reviewed relevant literature and typical cases, and conducted a holistic analysis to comprehensively identify and summarize common adverse event types and mechanisms in juvenile patients using cardiac assist devices, and to explore mitigation and prevention pathways.

To understand the major adverse events, this study retrieved data on adverse events related to the use of cardiac assist devices in juvenile patients from the US FDA’s MAUDE (Manufacturer and User Facility Device Experience) database from 2015 to 2025. Information on adverse events related to juvenile patients was extracted, including the time of occurrence, device type, complication manifestations, event outcomes, and potential triggers. This study systematically collected clinical research, retrospective analyses, registry studies, and review articles published in the past 10 years related to the use of cardiac assist devices in children and adolescents. The focus is on the types and reporting trends associated with various device types (IABP, ECMO, VAD); the mismatch between the child’s body size, physiological characteristics, and the device; the technical difficulties and nursing challenges in device management; and typical case analyses leading to death or serious injury. It also incorporated typical cases of adverse reactions encountered in pediatric hospitals using VAD, ECMO, and IABP support therapy. Through case comparison and analysis, high-risk points in device use and potential technical or nursing loopholes will be identified. Finally, the study will explore ways to mitigate these adverse reactions.

Table 1 details the core information and data extraction strategies for the three types of medical devices, focusing on data retrieval, filtering, and cleaning. Currently, specialized models exclusively designed for pediatric use are lacking for both IABP and ECMO devices. Consequently, following the retrieval of data from the MAUDE database, these devices are precisely identified and categorized based on pediatric-specific dimensions—such as balloon capacity and catheter outer diameter—as well as dedicated pediatric brands and model names. It should be noted that while classification based on device-specific physical dimensions (such as cannula French sizes for ECMO) represents the clinical ideal, the unstructured narratives in the MAUDE database systematically lack these dimensional parameters. Thus, categorizing ECMO devices based on core brand sequences and models serves as a necessary and practical surrogate strategy in this study. In contrast, VADs possess distinct boundaries regarding patient populations. In this study, the Berlin Heart is explicitly classified under the pediatric group, whereas devices commonly utilized in adults, such as the HeartMate series, are categorized under the adult group.

**Table 1:** Retrieval and Data Cleaning Strategies.

Product Classification	Product Code	FDA Receipt Date	Data Extraction Criteria
IABP	DSP	01/01/2015–01/01/2025	Pediatric: Balloon capacity 2.5cc – 20cc; Catheter outer diameter 5Fr – 7Fr. Adult: Balloon capacity 25cc – 50cc (commonly 30/40/50cc); Catheter outer diameter 7.5Fr – 8Fr.
ECMO	DTZ	01/01/2015–01/01/2025	Categorization based on core brands and model sequences.
VAD	DSQ	01/01/2015–01/01/2025	Pediatric: Berlin Heart. Adult:HeartMate series.

During the data cleaning phase, invalid records within the “Patient Problems” field that were explicitly labeled as “information not available/unknown” or characterized by a significant lack of descriptive detail were excluded. Following the aforementioned screening and cleaning procedures, the final sample size included in the analysis is as follows: the pediatric group comprises 97 VAD reports, 122 ECMO reports, and 61 IABP reports; the adult group consists of 500 VAD reports, 316 ECMO reports, and 415 IABP reports.

## Results

### Comparison of IABP, VAD and ECMO

**Fig 1:**
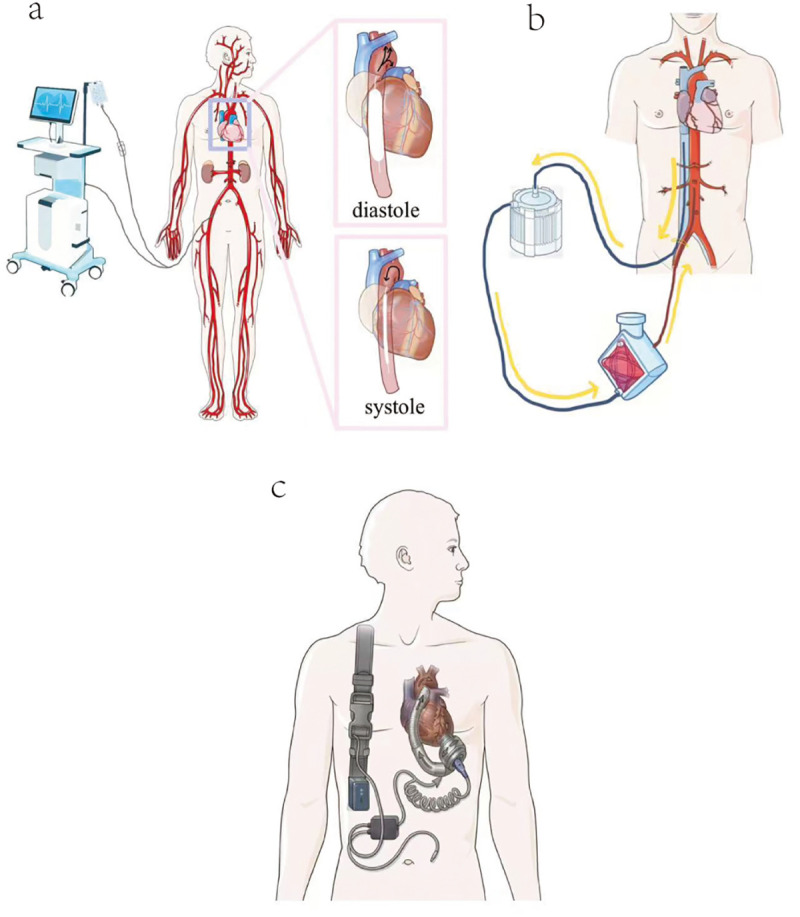
Mechanical circulatory support devices. (a) Working principle diagram of IABP. (b) Working principle diagram of ECMO. (c) Working principle diagram of VAD.

During the cardiac cycle to temporarily reduce left ventricular load and improve coronary blood supply. Using IABP can not only reduce the incidence of intraoperative hypotension and no-reflow after recanalization of occluded vessels, but also reduce the possibility of myocardial ischemia-reperfusion injury [4].It is suitable for some larger adolescent patients, but its application is limited in infants and young children, and it is mainly used for short-term circulatory support and postoperative assistance. ECMO is a device that can replace breathing in the short to medium term and has the ability to assist both ventricles at the same time. It is usually used for patients with heart failure of the whole heart and hypoxic lung disease with weak spontaneous breathing. In children, it is often used for severe cardiopulmonary failure, postoperative cardiac surgery or severe ARDS, and is an important means of rescuing critically ill children [5]. VAD is a device mainly used for medium to long-term mechanical circulatory support. It can partially or completely replace the pumping function of the ventricle and is usually used for patients with heart failure of one or two ventricles. Ventricular assist devices (VADs) can assist the left ventricular (LVAD), right ventricular (RVAD), or biventricular (BiVAD) systems, helping to maintain continuous blood flow to the body or lungs, thereby sustaining organ perfusion and vital functions. They are particularly suitable for children with end-stage heart failure who require bridging heart transplants or are awaiting recovery. The selection of a VAD must strictly consider the child’s weight, age, and anatomical conditions, and is suitable for some medium to large-sized children or specially designed devices for infants and young children [6]. Fig 1 shows the working principles of three types of equipment. Table 2 below provides a detailed comparison of the core parameters of these three types of devices when used by pediatric patients.

**Table 2:** Comparison of Core Parameters.

	IABP	VAD	ECMO
**Applicable age/weight**	older teenagers	3 kg+	All age groups
**Anatomical limitations**	Small aortic diameter → high risk	The size of the patient needs to be matched.	No strict restrictions
**Main indications**	Short-term low heart output	End-stage heart failure, bridging transplant/recovery	Cardiogenic shock, respiratory and circulatory failure
**Support duration**	Several days to 1 week	Weeks to months (long term)	Several days to 2 weeks (short term)
**Anticoagulation requirements**	Low-to-medium (heparin)	High (complex, requires monitoring of INR/ACT)	High (systemic heparinization)
**Pediatric use frequency**	less	More widely	acute and critical illnesses

### Comparison of IABP, VAD and ECMO

#### Analysis of adverse event reports for pediatric patients.

To further understand the actual occurrence of adverse events related to cardiac assist devices (CADs) in underage patients, this study systematically searched and analyzed the FDA’s MAUDE (Manufacturer and User Facility Device Experience) database.

MAUDE is a public database used by the FDA to collect medical device adverse event reports (MDRs). Dating back to 1991, it includes mandatory reporting from manufacturers and user facilities, as well as voluntary reporting from healthcare professionals and patients. MDR reports submitted within the last 10 years can be queried through the main interface, and the data is updated monthly. Earlier data can still be downloaded from the FDA’s MDR data files.

VAD devices are the only category with multiple HDE approval records (these devices are typically used for rare diseases or rare conditions such as pediatric heart failure), primarily focusing on pediatric or short-term mechanical circulatory support. This primarily includes DeBakey VAD Child (hereinafter referred to as HeartAssist5 Pediatric VAD), Berlin Heart EXCOR Pediatric Ventricular Assist Device, and Impella RP. Among them, the Berlin Heart EXCOR VAD is specifically designed for children and has the highest usage rate. Therefore, its sample data is analyzed as an example.

**Fig 2:**
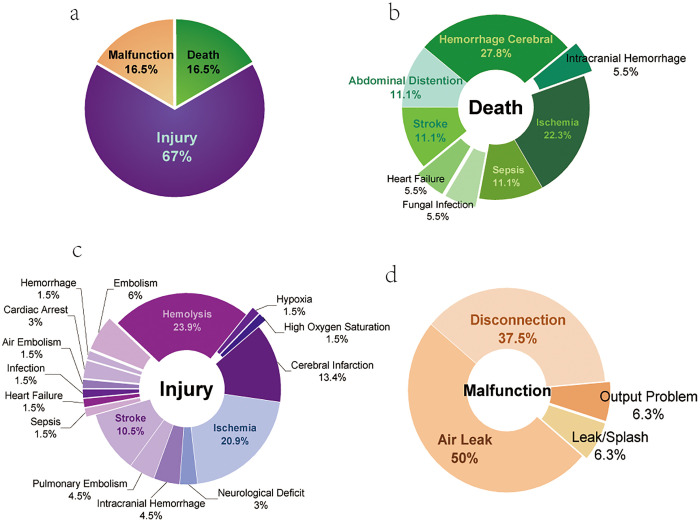
The adverse event report of VAD used in pediatrics in FDA database. (a) The percentage of all adverse events resulting from equipment failure, patient death, and injury to patients. (b) The percentage of all adverse events resulting in death, including all complications. (c) The percentage of all complications causing injury to patients. (d) Percentage of adverse events resulting from equipment failure.

From the MAUDE database, 97 adverse event reports from the past 10 years (January 2015 to January 2025) were extracted (serious events leading to death accounted for 16.5%, device malfunction accounted for 16.5%, and harm to patients accounted for 67%). Neurological complications were prevalent in fatal events (27.8% of deaths were due to cerebral hemorrhage, and 5.5% to intracranial hemorrhage), suggesting that brain-related complications may be a significant cause of death. Air leaks (50%) and disconnections (37.5%) were the two most common equipment failures, which can be attributed to mechanical damage, reflecting potential design flaws in the equipment’s mechanical connections or seals, or challenges in operation and maintenance. Hemorrhagic injuries (4.5% intracranial hemorrhage + 1.5% hemorrhage) totaled 6%, ischemic injuries (cerebral infarction 13.4% + stroke 10.5% + ischemia 20.9% + pulmonary embolism 4.5%) totaled 49.3%, acute organ injuries (cardiac arrest 3% + heart failure 1.5%) totaled 4.5%, and neurological dysfunction (3%). The significant imbalance between ischemic and hemorrhagic injuries suggests that anticoagulation management strategies urgently need improvement Fig 2.

Most ECMO devices still use adult-sized systems, but have been optimized for use in children. A small number of dedicated pediatric devices also exist, such as Medtronic’s Affinity series oxygenators for newborns/infants and Terumo’s FX05 pediatric model.

**Fig 3:**
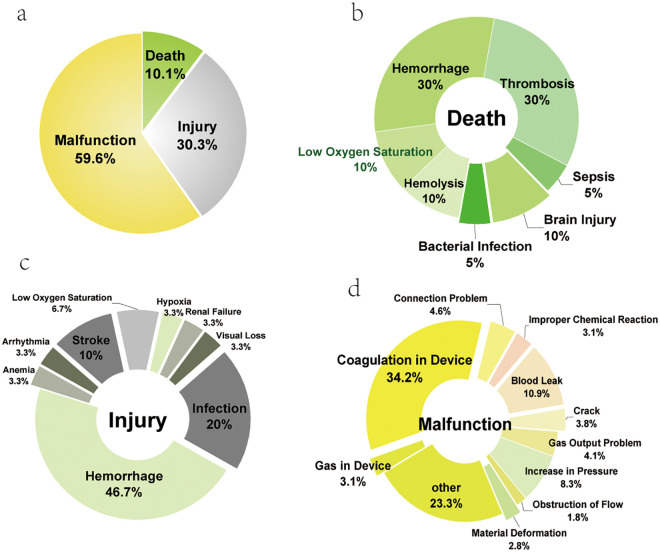
The adverse event of ECMO used in pediatrics in FDA database. (a) The percentage of all adverse events resulting from equipment failure, patient death, and injury to patients. (b) The percentage of all adverse events resulting in death, including all complications. (c) The percentage of all complications causing injury to patients. (d) Percentage of adverse events resulting from equipment failure.

In the application of ECMO in pediatric patients, we extracted data from 122 pediatric patients based on the product names and models. Analysis revealed that adverse events accounted for 10.1% of deaths. Hemorrhage and thrombosis were the two most frequent complications; among various injuries, hemorrhage was the leading cause, accounting for 46.7%. Brain injury, renal failure, and vision loss each occurred in 3.3% of cases. Furthermore, functional problems with the device itself were significant, with intra-device coagulation being the most prominent, accounting for 34.2% of all reports. This was followed by blood leakage and increased internal pressure, with incidence rates of 10.9% and 8.3%, respectively. Connection problems and abnormal gas output also accounted for a certain proportion, at 4.6% and 4.1%, respectively Fig 3.

Given the high risks mentioned above, future applications of pediatric ECMO should focus on anticoagulation management to reduce the incidence of thrombosis and bleeding, and closely monitor the functional status of the device.

Compared to VAD and ECMO, IABP is not widely used in pediatrics and has a small market. However, considering clinical needs, small balloon catheters, typically less than 34cc, have been designed specifically for children based on balloon volume and catheter size. Therefore, reports of adverse events related to this type of device are not sufficiently detailed.

**Fig 4:**
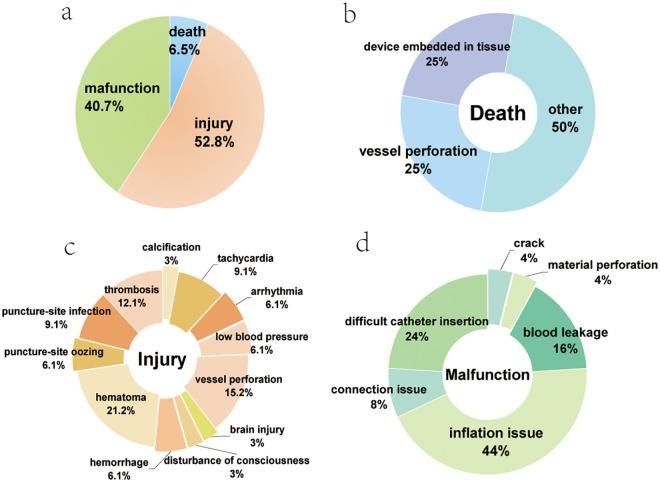
The adverse event report of IABP used in pediatrics in FDA database. (a) The percentage of all adverse events resulting from equipment failure, patient death, and injury to patients. (b) The percentage of all adverse events resulting in death, including all complications. (c) The percentage of all complications causing injury to patients. (d) Percentage of adverse events resulting from equipment failure.

Among the 61 extracted reports, device malfunction and patient injury were the most prevalent, accounting for 40.7% and 52.8% respectively, with deaths resulting from these events accounting for 6.5%. Among physiological complications, thrombosis (12.1%) and tachycardia (9.1%) were the main problems. Neurological events (such as brain injury and altered consciousness) occurred at a low rate (both 3%). The unique nature of IABP also results in its distinctive adverse event spectrum, including a high incidence of vascular complications and balloon inflation problems. Among vascular-related complications, device embedding and vascular perforation were the most common (each accounting for 25%). Puncture site complications (such as hematoma, bleeding, and infection) accounted for over 36% of all cases Fig 4.

The results indicate that the application of IABP in pediatrics faces unique risks of vascular injury. It is necessary to pay close attention to the risks of puncture and thrombosis caused by the physiological characteristics of children, and to be aware of device malfunctions, especially balloon inflation issues.

**Fig 5:**
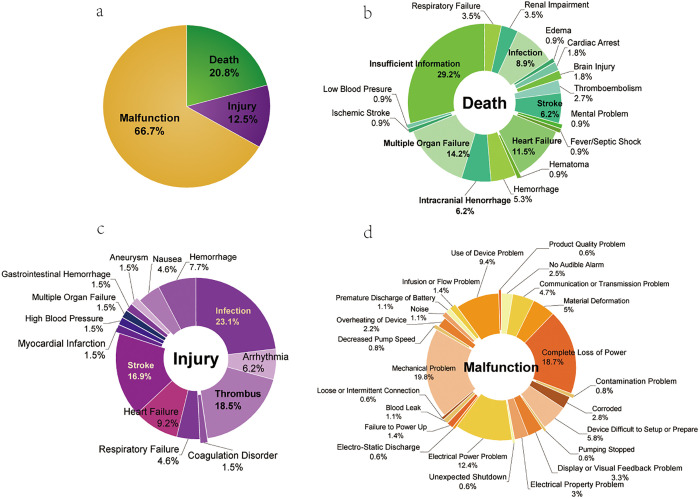
The adverse event report of VAD used in adults in FDA database. (a) The percentage of all adverse events resulting from equipment failure, patient death, and injury to patients. (b) The percentage of all adverse events resulting in death, including all complications. (c) The percentage of all complications causing injury to patients. (d) Percentage of adverse events resulting from equipment failure.

**Fig 6:**
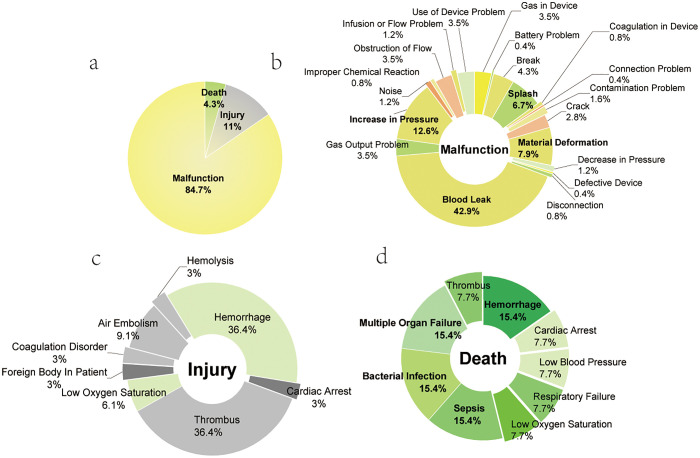
The adverse event report of ECMO used in adults in FDA database. (a) The percentage of all adverse events resulting from equipment failure, patient death, and injury to patients. (b) Percentage of adverse events resulting from equipment failure. (c) The percentage of all complications causing injury to patients. (d) The percentage of all adverse events resulting in death, including all complications.

**Fig 7:**
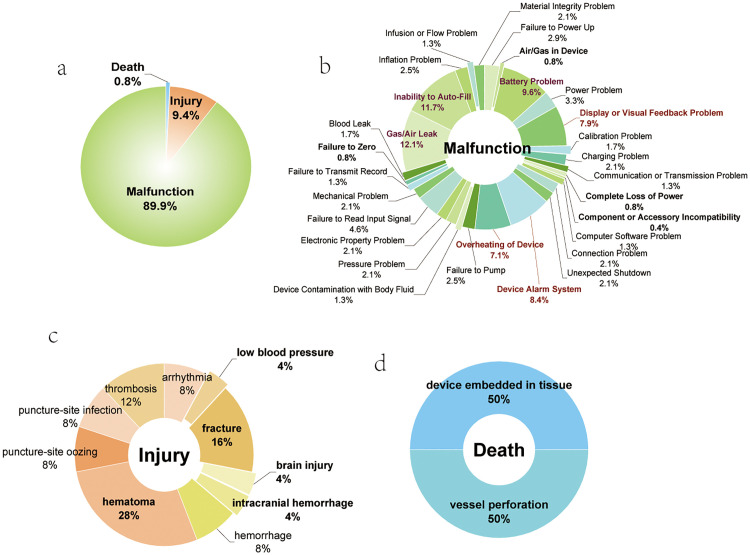
The adverse event report of IABP used in adults in FDA database. (a) The percentage of all adverse events resulting from equipment failure, patient death, and injury to patients. (b) Percentage of adverse events resulting from equipment failure. (c) The percentage of all complications causing injury to patients. (d) The percentage of all adverse events resulting in death, including all complications.

To further investigate the differences in adverse events during the use of the three types of devices between adults and pediatrics, we extracted data from 500 adult patients using VAD, 316 cases of ECMO, and 415 cases of IABP. The specific incidence rates of adverse events are shown in Figs 5, 6 and 7. Compared to adverse events observed in adults using the three types of cardiac assist devices mentioned above, pediatric patients exhibit characteristics of high injury rates and low device failure rates.Because any injury to a child’s organs can affect their future growth, clinical attention is more focused on adverse events in pediatric patients, and doctors define the scope of injury more broadly. This also reflects the inherent limitations of the MAUDE database—reporting and identification bias. Furthermore, adults have a relatively mature cardiovascular system compared to pediatric patients, which increases compatibility with the devices. Conversely, in pediatric patients, especially infants, the cardiovascular system is still developing, and changes in flow or pressure caused by the device can quickly lead to acute end-organ damage, such as renal failure and acute mesenteric ischemia, which are complications caused by hemodynamic disturbances.

In cardiovascular diseases, most of these devices are designed for adults, and the mechanical wear and tear from long-term use increases the risk of device failure during use. However, because clinical experience in managing device use in adults is more extensive than in pediatrics, abnormalities can be adjusted promptly to minimize harm. Therefore, it exhibits the characteristics of high device failure rates and low injury rates. Increasing the safety and reliability of the devices is therefore crucial.

#### Comparison of device use risks for adults and children.

Based on traditional database analysis, we then systematically collected clinical studies, retrospective analyses, registration studies and review articles published in the past 15 years related to the use of cardiac assist devices in children and adolescents as references to compare and analyze the differences in six major types of complications that occur in adult and pediatric patients during the use process. (Including vascular complications, bleeding, infection, nervous system injury, thrombosis, and device-related complications)

**Fig 8:**
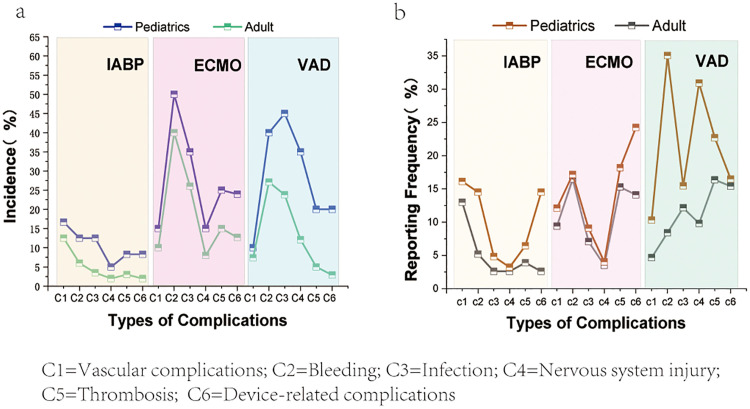
Comparative analysis of risk profiles between adult and pediatric populations. (a) Comparison of the incidence of adults and children from literatures.(b)Comparison of the reporting frequency of adults and children based on Maude datebase.

While both Fig 8a and 8b (Fig 8) reveal that the incidence of adverse events is generally higher in juvenile patients than in adult patients when using IABP, ECMO, and VAD. This does not imply that either analysis is “wrong,” but rather reveals the fundamental difference between real-world spontaneous reporting systems and rigorous clinical/registry studies.This difference is systematic and primarily stems from the nature of the data sources. The first figure, from a clinical study, reflects the true biological risk of complications observed under ideal, controlled conditions; while the second figure, from the MAUDE database, reveals the tendency of device problems identified and reported in broad clinical practice due to various factors.

The total sample size of the adult and pediatric groups (1231 vs. 280) presents a potential risk of bias; therefore, we conducted a multi-dimensional statistical evaluation using SPSS and Microsoft Excel. Based on the reporting frequency and counts of associated complications, Fisher’s Exact Test performed in SPSS confirmed a highly significant difference in the distribution of complications between pediatric and adult patients within the VAD group (*P* < 0.001). This significance, established while accounting for the imbalance in sample sizes, demonstrates that pediatric risks are not merely a scaled version of adult data and cannot be directly extrapolated from adult clinical findings.

Furthermore, to identify the risk enrichment intensity of specific complications and mitigate systemic reporting bias, the Proportional Reporting Ratio (PRR)—a common metric in regulatory science—was introduced for risk signal detection. The calculation results (see Table 3) indicate that the PRR values for various device complications exhibit a non-homogeneous distribution, encompassing both strong risk signals (*PRR* > 2.0) and numerous items with *PRR* < 1.0. This unbalanced distribution pattern reflects that the MAUDE database records objective clinical risk disparities rather than systemic biases arising from differing reporting habits between groups.

**Table 3:** Fisher’s Exact Test and PRR Test Results.

Device Type	*P*-Value (Fisher)	Complication Type	PRR
IABP	0.144 (NS)	C1	0.63
		C2	1.38
		C3	0.92
		C4	0.61
		C5	0.84
		C6	2.76
ECMO	0.809 (NS)	C1	1.00
		C2	0.81
		C3	1.00
		C4	0.91
		C5	0.92
		C6	1.33
VAD	< 0.001	C1	1.14
		C2	2.13
		C3	0.65
		C4	1.61
		C5	0.71
		C6	0.55

Note: NS denotes non-significant (P > 0.05).

For the IABP and ECMO groups, although the P-values from the exact tests did not reach statistical significance, the PRR for device-related issues (c6) in the IABP group reached 2.76. While this preliminary signal offers potential regulatory early-warning insights, it must be interpreted with caution as a hypothesis-generating finding due to the small sample size. In contrast, the ECMO group displayed a relatively stable overall distribution, with PRR values across various dimensions fluctuating around 1.0, suggesting a high degree of consistency in risk patterns across different populations. To a large extent, the lack of statistical significance in these groups is attributed to the inherent scarcity of pediatric clinical data and the resulting smaller sample size.

### Adverse event spectrum

The use of IABP in juvenile patients has a relatively high incidence of related adverse reaction events due to its anatomical and physiological characteristics. It mainly includes vascular complications [7–9] (such as limb ischemia, arterial occlusion, and edema at the puncture site), bleeding events [10,11] (especially bleeding at the puncture site and retroperitoneum), infections [12,13] (infections related to the puncture site and catheter), and device failures [14]. In addition, some children may also experience thromboembolic events [15] (such as stroke), hemolysis and coagulation disorders [16,17], ischemia of the kidneys and mesentery [18], and neurological complications [9,19]. Table 4 below shows the main adverse events that occurred in the application of pediatric IABP.

**Table 4:** Adverse events in the application of IABP in pediatrics.

Types	Specific characteristics	Potential causes	High-risk factors	Incidence
Vascular complications	Femoral artery occlusion, limb ischemia, hematoma at the puncture site.	Large duct diameter, thin vessel wall, and thrombosis after damage to the arterial intima.	Low body weight, poor arterial development, and inadequate puncture technique.	20%−40%
Bleeding complications	Continuous bleeding from the puncture site, retroperitoneal hemorrhage, intracranial/gastrointestinal bleeding.	Excessive anticoagulation, thrombocytopenia, and impaired coagulation function.	Newborns, inadequate anticoagulation monitoring, coagulation disorders.	10%−25%
Infectious complications	Puncture site infection, catheter-related sepsis.	Prolonged catheter indwelling time, improper local care, and skin barrier damage.	Weakened immunity, catheter contamination, inadequate nursing care.	5%−15%
Thromboembolism	Intraballoon thrombosis, distal arterial embolism, stroke pulmonary embolism.	Catheter foreign body reaction, insufficient anticoagulation, blood flow disturbance.	Hypercoagulable state, inadequate anticoagulation, rough catheter surface.	5%−15%
Catheter displacement	Balloon displacement, slip, exposed, counterpulsation function failed,synchronization, drive air pump malfunction.	Frequent movement, poor fixation, abnormal catheter tension.	Infants, agitated patients, and those lacking restraint devices.	10%−20%
Hematological complications	Hemolysis,low platelets,DIC,anemia.	High shear forces damage red blood cells and disrupt blood clotting function.	Prolonged counterpulsation, high-intensity counterpulsation, abnormal anticoagulant response.	5%−10%
Renal/Mesenteric Ischemia	Decreased urine output,acute kidney injury, bloating,intestinal necrosis.	Inadequate aortic perfusion, distal blood flow obstruction, microvascular embolism.	Aortic dissection, high catheter position, and low perfusion status.	Case Report
Neurological complications	Stroke, epilepsy,disorders of consciousness cognitive impairment	Thrombus detachment, insufficient cerebral perfusion, hemorrhage.	Young age, history of encephalopathy, anticoagulation imbalance.	2%−8%

VAD is mainly used in children to bridge heart transplants or for long-term support while waiting for the recovery of heart function. Thrombosis [20] and stroke [21] are the most threatening adverse events of VAD, often involving the central nervous system. In addition to stroke, non-specific brain injury and neurodevelopmental delay caused by unstable perfusion also cause damage to the nervous system of children, especially in children under one year old, which requires high vigilance. Bleeding events are closely related to long-term anticoagulant therapy and are particularly common in the intracranial and digestive tracts [22]. Driver-related infections [23] are also more common complications with longer use, especially at the connection between the skin puncture site and the pump body. Hemolysis of the device is manifested as anemia, elevated bilirubin, and even acute kidney injury due to the high-speed rotation of the pump or excessive shear force inside the pump [23]. Table 5 below shows the main adverse events that occurred in the application of pediatric VAD.

**Table 5:** Adverse events in the application of VAD in pediatrics.

Adverse reaction types	Specific characteristics	Potential mechanisms or causes	High-risk factors	Incidence
Bleeding complications	Gastrointestinal bleeding, intracranial hemorrhage, bleeding at the puncture site	Excessive anticoagulation, thrombocytopenia, and vascular shearing caused by VAD itself.	Inappropriate anticoagulant dosage, liver dysfunction, age under 5 years.	15%−35%
Infectious complications	Drive line port infection, pump cavity infection, Septicemia.	The drive wire penetrates the skin to form an open channel for long-term placement of the device.	Used for more than 30 days, inadequate care, poor nutritional status.	20%−40%
Thrombotic complications	Cerebral infarction, intrapump thrombosis, pulmonary embolism, limb embolism.	High shear at the blood-pump interface, insufficient anticoagulation, and improper pump shutdown.	Small body weight, inadequate anticoagulation, and unstable equipment operation.	10%−30%
Hemolytic Complications	Hemoglobinuria, jaundice, anemia.	High shear forces inside the pump cause red blood cells to rupture and the device to operate unstably.	High-speed operation, small pump chamber with large flow rate, young age group.	10%−25%
Neurological complications	Stroke, epilepsy, confusion, cognitive developmental disorders.	Thrombus detaches and enters cerebral circulation, cerebral insufficiency, intracranial hemorrhage.	Young children, history of stroke, bleeding tendency.	10%−20%

ECMO is widely used in pediatrics for the rescue of acute cardiopulmonary insufficiency. The most common adverse event is hemorrhagic complications [24], including intracranial hemorrhage, incision bleeding and gastrointestinal bleeding. Newborns are particularly susceptible. Thrombotic and embolic events often result from unstable blood flow in membrane oxygenators or circuits, which can develop into stroke or limb ischemia [25]. Due to long-term catheter indwelling and immature immune system development, the risks of sepsis, skin and pulmonary infections, and catheter-related bloodstream infections also significantly increase [26]. In addition, hemolysis [27] and renal function impairment [28] are a typical combination of complications. The destruction of red blood cells generates free hemoglobin, which aggravates the damage to renal tubules. Neurological complications include cerebral edema, convulsions, and delayed developmental disorders, often caused by perfusion fluctuations or small thrombecs and microthrombecs. Some patients, even if they survive, have medium – and long-term cognitive dysfunction [29]. Table 6 below shows the main adverse events that occurred in the application of pediatric ECMO.

**Table 6:** Adverse events in the application of ECMO in pediatrics.

Adverse reaction types	Specific characteristics	Potential mechanisms or causes	High-risk factors	Incidence
Bleeding complications	Intracranial hemorrhage,bleeding at the insertion site,gastrointestinal bleeding,hemothorax.	Anticoagulation therapy,immature coagulation function,fragile blood vessels, and procedural trauma.	Newborns, those on long-term anticoagulation therapy, and those with coagulation disorders.	30%–50%
Infectious complications	Catheter-related bloodstream infection, lung infection, skin infection, septicemia.	Prolonged intubation, low immunity, and improper aseptic technique.	Indwelling time over 7 days, insufficient antibiotic coverage, newborns.	15%–25%
Thrombotic complications	Oxygenator thrombosis, intra-catheter thrombosis, stroke, pulmonary embolism.	Insufficient anticoagulation, stagnant flow in pipelines, and hypercoagulable state.	Small body weight, small catheter diameter, abnormal pump rate.	10%–20%
Hemolytic Complications	Hemolysis, DIC, thrombocytopenia, jaundice, urine discoloration.	High flow rates lead to mechanical damage to red blood cells and disruption of anticoagulation balance.	High pump rate, low blood volume state, hypothermia.	15%–30%
Neurological complications	Intracranial hemorrhage, cerebral infarction, convulsions, coma, developmental delay.	Thrombosis or hemorrhage leading to abnormal cerebral perfusion, neonatal cerebral hemorrhage.	Young age (under 1 year), high pump rate/high pressure, bleeding constitution.	10%–30%
Kidney damage	Oliguria, anuria, CRRT required, azotemia, acidosis.	Hypoperfusion status, hemolytic product load, and uneven ECMO perfusion.	ECMO over 7 days, low birth weight, and concurrent infection.	20%–40%
Mesenteric ischemia/gastrointestinal dysfunction	Bloating, intestinal necrosis, intestinal bleeding, paralytic ileus.	Blood flow preferentially perfuses the brain/heart, with insufficient perfusion to the mesentery.	Prolonged low blood pressure, blood flow redistribution, intestinal infection.	5%–10%

## Disscusion

### Pre-maket regulation from a strandard point

#### Most challenged parts of VAD test clauses standard.

Most adverse event reports in the MAUDE database are submitted by manufacturers rather than clinicians. So they focus more on equipment-related adverse events rather than clinical complications. Therefore, in the above data analysis process, it can be found that the proportion of equipment failures is relatively high. To further investigate the problems existing in the post-marketing regulatory system, we mapped these faults that occurred during patient use to relevant detection standards and studied the distribution of faults exposed after marketing.

**Fig 9:**
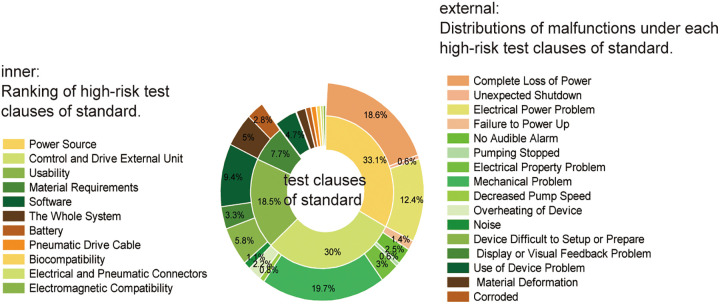
Distribution of equipment failures in adult patients using VAD in the main test chapter of ISO14708-5/YY-T 0989.5.

**Fig 10:**
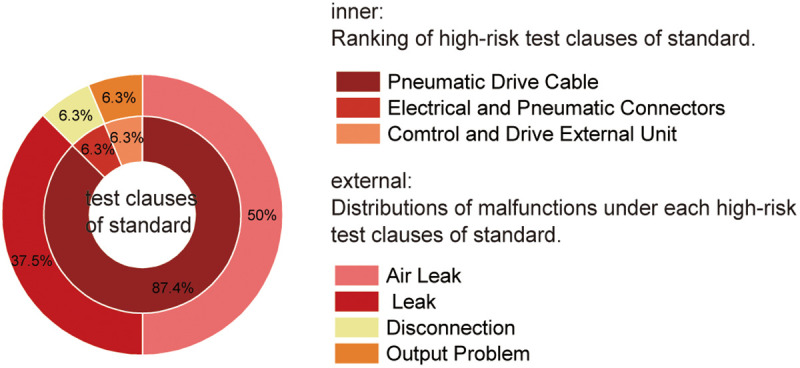
Distribution of equipment failures in pediatric patients using VAD in the main test chapter of ISO14708-5/YY-T 0989.5.

According to the data, in adult VADs, the majority of delays are related to 110V power source (33.1%) and control and external unit (30%), with human factor accounting for 18.5% Fig 9. In pediatric VADs, the delay risk associated with pneumatic drive cables is as high as 87.4%, mainly due to adverse events such as gas leaks and fluid splashes Fig 10. Based on a comprehensive analysis of both data sets, control and external unit present a significant risk of delays during pre-market testing. It can be seen from this that these two standards do not pay sufficient attention to the risk points exposed after listing, and there is a lag in supervision. It is urgent to update them in a timely manner based on the real non-performing data.[30,31] Therefore, it is recommended to strengthen product reliability testing; extend the follow-up period of clinical trials, requiring manufacturers to provide clinical data promptly, focusing on reliability, repair rates, and serious adverse events related to the controller, to enable dynamic monitoring; and simultaneously utilize early real-world data from high-quality registry systems to supplement traditional clinical trials, allowing for timely risk identification and device improvements.

#### Survy on researchers and manufacture’ Cognition of risk based on VAD standard.

**Fig 11:**
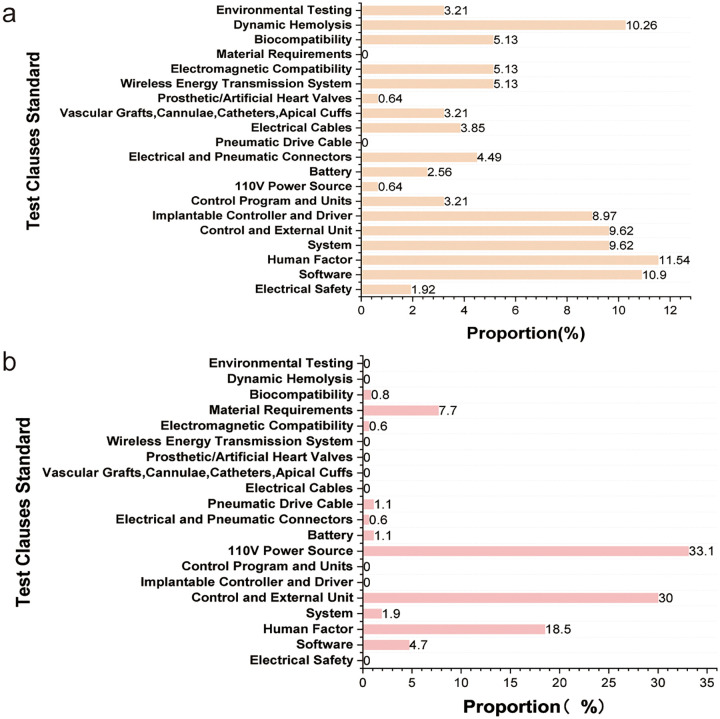
Survy on Researchers and manufacture’ Cognition of risk based on VAD standard. (a) Investigation into the manufacturer’s understanding of the device’s potential risks based on VAD test clauses standard. (b) Chapter distribution of device’s potential risks identified based on MAUDE data.

In response to the risks exposed by patients during the post-marketing use of VAD in the database, we conducted a questionnaire survey on the awareness of device risks among 7 Chinese manufacturers. Based on the results of 52 collected questionnaires, the statistics are shown in Fig 11a Fig 11. And compare the results with the device risks of VAD in the MAUDE database (as shown in Fig 11b) Fig 11. The results show that most manufacturers believe that the testing items such as human factor, software, dynamic hemolysis, control and external unit, and system are most likely to have a lag risk. However, according to the analysis of the MAUDE database, the three testing items of 110V power source, control and external unit, and human factor have a relatively large lag risk. Therefore, in response to the lag risk, based on the understanding of manufacturers and the results of the database, the current two detection standards for VAD do not pay sufficient attention to the risk points exposed after going on the market. It is urgent to make updates and adjustments according to the actual poor data. This will enhance the safety of the equipment usage process and further ensure the life safety of pediatric patients.

### Post-maket regulation of VAD

#### Post-market evidence collection mechanisms for medical devices in the US, Europe, and China.

After a device is launched on the market, it is equally important to continuously and systematically collect data evidence related to its safety, effectiveness, and performance in real-world clinical use environments. This evidence can verify and monitor the long-term performance of the product in practical applications, identify and manage potential risks, accumulate real-world evidence, and support scientific decision-making throughout the product lifecycle [32].

The U.S. FDA implements a conditional approval mechanism for high-risk medical devices. After granting market approval based on preliminary evidence, it mandates that manufacturers, distributors, healthcare providers, and patients report adverse events and may require post-market approval studies to continuously collect clinical data to support long-term safety and efficacy assessments. The European Union, under the MDR requirements, after granting a CE mark to a product, and in cases where evidence is insufficient but the product has potential clinical value, the notified body may require manufacturers to enter information such as clinical studies, post-market clinical follow-up studies (PMCF) [33], adverse events, and corrective actions into the EUDAMED database to enhance transparency and support continuous monitoring. China (NMPA) implements a conditional approval system for medical devices, requiring manufacturers and users to report serious adverse events within 5 days and mandating post-market studies to continuously verify safety and effectiveness, submitting all relevant data to the NMPA database. Meanwhile, China is conducting regulatory technology research on the preclinical safety evaluation of VAD from the perspectives of experimental techniques and evaluation methods [34–36]. Although their market approval pathways differ significantly, all three systems emphasize post-market evidence collection to ensure that regulatory agencies can continuously monitor product risks [37–39].

#### Discussion on real-time AI regulatory strategies for mitigating pediatric device risk latency.

At the signal detection layer, regulatory bodies could deploy NLP-based automated text mining pipelines against unstructured adverse event narratives in databases such as MAUDE. This direction has precedent in pharmacovigilance research: combining FAERS spontaneous reports with NLP-processed electronic medical records has been shown to substantially increase the recall of adverse drug event signals compared with either source alone [40], and a 2025 scoping review of seven independent studies confirmed that NLP/machine-learning techniques applied to unstructured clinical text can detect under-reported adverse events and safety signals not apparent from structured data alone, although it also noted substantial heterogeneity in methods and a lack of standardized validation criteria across studies [41]. Domain-adapted language models—fine-tuned on medical device failure terminology—could in principle enable continuous disproportionality analysis through dynamic PRR tracking, extending this logic to high-acuity device signals such as hemorrhagic complications in pediatric VAD populations or mechanical failure modes in IABP systems; however, this specific application has not yet been validated and should be regarded as a proposed direction rather than a demonstrated capability.

At the evidence synthesis layer, in silico clinical trials and digital twin modeling offer a more concrete precedent. The FDA’s own Center for Devices and Radiological Health has published a formal credibility-assessment workflow for in silico clinical trials of medical devices, explicitly intended to support regulatory evaluation of device safety and effectiveness using computational models of patients [42]. A related hierarchical framework for establishing credibility in medical device in silico trials has also been proposed to address key validation challenges [43]. More broadly, in silico trials and digital twins are increasingly recognized across regulatory science as tools that can supplement or, in some cases, substitute for traditional clinical trials, including for rare or vulnerable populations where conventional trial recruitment is difficult [44]—a description that applies directly to pediatric cardiac assist device cohorts. Building on this precedent, key parameters extracted from pediatric MAUDE adverse event reports could in principle be propagated into computational simulation environments such as the physiological control algorithm frameworks examined in this study, enabling prospective in silico verification of proposed corrective measures prior to clinical implementation. This remains a conceptual extension, however, and would require dedicated validation work specific to pediatric mechanical circulatory support before it could inform regulatory decisions.

At the regulatory response layer, signal detection and in silico evidence could be coupled with existing standards frameworks (e.g., YY/T 0989.5) through systematic mapping between identified failure patterns and corresponding normative clauses, feeding into a regulatory sandbox mechanism that issues interim technical guidance ahead of formal standard revision. Regulatory sandboxes of this kind are already an established adaptive-regulation tool, and recent work has specifically examined extending sandbox models from fintech into medical artificial intelligence, highlighting both the potential to accelerate safe market access and the risks—data privacy, real-world validation gaps, and the need for robust post-sandbox surveillance—that must be managed when applying this model to healthcare technologies [45]. Extending such a sandbox model to physical implantable devices such as pediatric VADs, rather than software-based medical AI, remains speculative and would require dedicated pilot studies before adoption.

### Limitations of the study

Furthermore, this study is based on the FDA’s MAUDE database, which serves as a typical passive surveillance system. As indicated by official FDA guidelines, the lack of total device implantation figures (the denominator), combined with the potential for under-reporting or duplicate reporting, precludes the use of MAUDE data for estimating the true clinical incidence of adverse events (AEs). Consequently, the data comparisons in this research should be interpreted as trend disparities in “reporting frequency” rather than a direct comparison of incidence rates.

Secondly, the asymmetry in data magnitude presents an additional challenge to this study. Due to the extremely low clinical utilization frequency of pediatric cardiac assist devices, the level of data detail in the pediatric group—particularly the IABP group (n = 61)—cannot achieve parity with the adult group, which may introduce statistical bias. Consequently, the insights derived from this small pediatric IABP sub-cohort should be strictly considered preliminary and hypothesis-generating rather than definitive conclusions. In the future, we will establish a continuous data collection mechanism to further validate these preliminary findings through long-term tracking and the integration of multi-center real-world data, thereby mitigating the sample bias inherent in passive surveillance systems.

Thirdly, the baseline data for the risk cognition assessment was derived from a targeted questionnaire administered to only 7 Chinese VAD manufacturers (*n* = 52 responses). Consequently, the generalizability of these survey insights to global VAD regulatory standards and international manufacturing paradigms may be limited due to potential geopolitical and localized institutional biases. Future prospective studies should include multicenter, internationally collaborative cohorts to cross-validate these perceptual risk discrepancies on a global scale.

Fourthly, our ECMO data screening relied primarily on specific brand lines and model sequences rather than device-specific physical dimensions. Because of the data omissions inherent in passive surveillance databases, this operational approach might introduce a potential risk of misclassification bias. Future medical device tracking registries should implement mandatory Unique Device Identification (UDI) barcoding to bridge this technical gap, ensuring that clinical adverse events are mapped by exact physical specifications rather than commercial branding.

## Conclusion

This study utilizes a 10-year retrospective analysis of the FDA MAUDE database to provide a descriptive overview of adverse event reporting trends for pediatric cardiac assist devices. The data reveal distinct descriptive differences in complication profiles between adult and pediatric populations, suggesting that adult safety signals may not be directly extrapolated to children.

On this basis, due to the characteristics of high device failure rate shown by adult data samples, in accordance with the ISO14708-5/YY-T 0989.5 detection standard, the real-world data of adult VAD were further analyzed to identify the risks that were not covered by the detection standard before going on the market.

However, given the inherent methodological boundaries of passive surveillance data and the small pediatric sample sizes, our findings must be characterized strictly as exploratory and hypothesis-generating. Rather than advocating for sweeping, immediate policy overhauls, this work highlights the conceptual merit of using post-market clinical trends to inform the iterative, data-driven optimization of medical device testing standards. Future extensive, multi-center active registries remain indispensable to confirm these preliminary signals and safely guide precise adaptive frameworks for pediatric populations.
